# Valproic acid and HIV-1 latency: beyond the sound bite

**DOI:** 10.1186/1742-4690-2-56

**Published:** 2005-09-19

**Authors:** Stephen M Smith

**Affiliations:** 1 Section of Infectious Diseases, Department of Medicine, Saint Michael's Medical Center and Department of Preventive Medicine and Community Health, The New Jersey Medical School, Newark New Jersey 07102, USA

## Abstract

A recent publication in *Lancet *by Dr. David Margolis and colleagues raised the prospect that HIV infection may be curable. In this pilot study, which received much attention from the press, Dr. Margolis'group found that valproic acid plus enfuvirtide reduces the pool of CD4^+ ^T-cells, which are latently infected with HIV-1, the so-called viral reservoir. This commentary critically addresses current data on this topic.

A recent publication in Lancet [1] has created quite a stir among HIV circles (no, not those containing 1 or 2-LTRs) [2] and the lay press. In a study led by Dr. David Margolis, four patients, each of whom had undetectable HIV plasma viral loads, had enfuvirtide and valproic acid (VPA) added to their anti-HIV regimens. The researchers measured several parameters with particular interest in the effect of VPA on resting CD4^+ ^T-cells latently infected with HIV-1. This pool of cells – often referred to as the viral reservoir – is thought to pose an significant obstacle to HIV eradication. Drs. Finzi, Siliciano, and colleagues first described these CD4^+ ^T-cells that produce little or no viral proteins but can be stimulated with mitogens to produce infectious virus [3]. This pool of cells is also thought to serve as a biological library for archival strains of drug-resistant HIV [4]. Even years after discontinuation of a given drug, resistant strains of HIV-1 quickly re-emerge when the drug is re-introduced into a therapeutic regimen. Therefore, in theory, a treatment protocol that reduces or eliminates this pool of infected cells could eliminate drug-resistant strains, and would have the potential to cure HIV infection. However, in the absence of effective drug intervention, there is little chance of eliminating this viral reservoir; the half-life and pool size of latently infected CD4^+ ^T-cells are considered too great to permit eradication under current regimens in most, if not all, patients. Dr. Siliciano and colleagues estimate the mean time to eradication is 51.2 years in the best case scenario [5], e.g., those patients who have undetectable viral loads and no viral "blips."

Dr. Margolis' laboratory previously demonstrated that VPA can stimulate the release of virus from latently infected CD4^+ ^T-cells *in vitro *[6]. The stimulatory effect of VPA is equal to, or greater than, that of the mitogen, PHA, but VPA has no effect on T-cell activation or virus production from mitogen-activated lymphoblasts. VPA inhibits histone deacetylase (HDAC)-1, which may be involved in suppressing HIV promoter activity in latently infected, resting CD4^+ ^T-cells. In the *Lancet *study, four patients with long-term, undetectable viremia were given enfuvirtide, an injectable HIV fusion inhibitor, added to their ongoing regimens. After 4–6 weeks, VPA was then started. The VPA dose (500–750 mg twice per day) was adjusted to maintain plasma concentrations within a defined range (50–100 mg/L). The frequency of infection in resting CD4^+ ^T-cells was measured twice at baseline prior to initiation of VPA therapy, and again 12 weeks after the start of VPA treatment. While baseline measurements showed little or no change in the frequency of latently infected CD4+ T-cells, enfuvirtide and VPA therapy decreased this measurement by 29–84% in all four subjects. No changes were observed in the frequency of HIV proviral DNA or immune activation markers. The authors conclude that HDAC inhibitors, such as VPA, could lead to HIV eradication when combined with other anti-HIV drugs.

The results of this pilot study are intriguing, but must be considered cautiously with a clear understanding of their inherent limitations. By design, the study was not controlled and each patient received two new drugs, enfuvirtide and VPA, and the relative contribution of each drug to lowering the frequency of latently infected CD4^+ ^T-cells is unknown. At least one group has demonstrated that intensification of anti-HIV therapy decreases the half-life of this population [7]. Moreover, absolute CD4^+ ^T-cell counts can vary significantly even in stable patients with undetectable viral loads, and this variability may influence quantitative assessments of the latent pool of infected cells. While the reported decrease in latently infected CD4^+ ^T-cells in the four patients receiving VPA is certainly promising, further evaluations of the efficacy of VPA in combination with other anti-HIV drugs will be needed in larger, controlled clinical studies.

This study also raises important issues regarding the use of enfuvirtide in an intensification regimen. As reported, two patients had residual viremia after intensification with enfuvirtide making it unclear whether this intensification is necessary or especially beneficial. Theses data suggest that intensification may not be necessary or helpful, or more importantly, that reduction of the reservoir pool may occur in the presence of on-going, low level viral replication. Presumably this issue will be addressed in future studies.

Of considerable interest is the potential mechanism by which VPA reduces the frequency of latently infected, resting CD4^+ ^T-cells. Presumably, through inhibition of HDAC, VPA allows initiation of viral transcription, which in turn leads to production of viral proteins and virions, and cell death due to virally induced cytotoxicity. Paradoxically, VPA does not activate resting CD4^+ ^T-cells, thus making it unclear how HIV transcription is upregulated and viral promoter activity is increased.

In this regard, many critical questions remain to be answered. Foremost, what happens to the pool of latently infected cells after enfuvirtide and VPA are discontinued? Data by Dr. Margolis and colleagues have shown VPA works quickly *in vitro *to induce virus production from latently infected cells. *In vivo*, very few new latently infected cells would be expected to develop over the duration of VPA treatment presented in their *Lancet *study. And yet a proportion of latently infected cells remained after 3 months of therapy. Do these remaining cells represent a distinct subset from those eliminated by enfuvirtide and VPA? Viral RNA clearance has two phases of decay [8]. Does the population of latently infected CD4^+ ^T-cells have similarly complicated kinetics?

Others have tried to reduce the latent viral reservoir, primarily through activation of resting CD4^+ ^T-cells. In one sobering example, Drs. Fauci, Chun, Lane and colleagues stopped anti-HIV medications in two patients, who had undetectable viral loads for years and had received IL-2 therapy [9,10]. At the time HIV therapy was discontinued virus could not be cultured from resting CD4^+ ^T-cells, either from the blood or lymph nodes, of either patient. More significantly, proviral DNA was below the limit of detection (0.5 copies per 10^6 ^PBMC). Despite these impressive laboratory findings, within three weeks, plasma HIV RNA levels became detectable and rose above 10,000 copies per ml.

Finally, the assumption that the population of latently infected, resting CD4^+ ^T-cells is the only reservoir for HIV-1 *in vivo *is largely untested. While the persistence of these cells likely guarantees chronic infection, their elimination may not result in eradication. Other pools of HIV-infected cells or tissue reservoirs may exist. While current results presented in the *Lancet *study certainly provide reason to be optimistic, it is critical to balance this optimism with further rigorous clinical evaluations, including larger, controlled studies of VPA and enfuvirtide. As always, the results of any pilot study must be interpreted with caution and placed in the proper context of existing knowledge. It is my hope that Dr. Margolis and colleagues are correct, and that HIV reservoirs can be eliminated through the additional administration of a small molecule, such as VPA. However, the challenge of eradicating HIV remains daunting, and history and science have yet to yield simple answers.
